# COVID-19 vaccination-related tinnitus is associated with pre-vaccination metabolic disorders

**DOI:** 10.3389/fphar.2024.1374320

**Published:** 2024-05-22

**Authors:** Weihua Wang, Anusha Yellamsetty, Robert M. Edmonds, Shaun R. Barcavage, Shaowen Bao

**Affiliations:** ^1^ Department of Physiology and Department of Otolaryngology—Head and Neck Surgery, University of Arizona College of Medicine, Tucson, AZ, United States; ^2^ Department of Audiology, College of Health and Human Sciences, San José State University, San José, CA, United States; ^3^ Catharus Scientific LLC, Tularosa, NM, United States; ^4^ Weill Cornell Medical College, New York, New York, United States

**Keywords:** SARS-CoV-2, COVID-19, vaccination, tinnitus, metabolic disease

## Abstract

Cases of tinnitus have been reported following administration of COVID-19 vaccines. The aim of this study was to characterize COVID-19 vaccination-related tinnitus to assess whether there is a causal relationship, and to examine potential risk factors for COVID-19 vaccination-related tinnitus. We analyzed a survey on 398 cases of COVID-19 vaccination-related tinnitus, and 699,839 COVID-19 vaccine-related reports in the Vaccine Adverse Effect Reporting System (VAERS) database that was retrieved on 4 December 2021. We found that following COVID-19 vaccination, 1) tinnitus report frequencies for Pfizer, Moderna and Janssen vaccines in VAERS are 47, 51 and 70 cases per million full vaccination; 2) the symptom onset was often rapid; 3) more women than men reported tinnitus and the sex difference increased with age; 4) for 2-dose vaccines, the frequency of tinnitus was higher following the first dose than the second dose; 5) for 2-dose vaccines, the chance of worsening tinnitus symptoms after second dose was approximately 50%; 6) tinnitus was correlated with other neurological and psychiatric symptoms; 7) pre-existing metabolic syndromes were correlated with the severity of the reported tinnitus. These findings suggest that COVID-19 vaccination increases the risk of tinnitus, and metabolic disorders is a risk factor for COVID-19 vaccination-related tinnitus.

## Introduction

The COVID-19 pandemic has caused social and economic stress worldwide. Although COVID-19 vaccines are effective in reducing symptomatic infection and symptom severity (Voysey et al., 2021; Mongin et al., 2023; Lam et al., 2024; Wu et al., 2024), they can lead to adverse effects in a small percentage of the vaccinated population. A possible adverse effect is tinnitus. Cases of tinnitus have been reported following COVID-19 vaccination (Parrino et al., 2021; Sadoff et al., 2021; Tseng, Chen, Sun, Chen and Chen, 2021; Canales Medina and Ramirez Gomez, 2022; Chen et al., 2022). Cases of tinnitus following COVID-19 vaccination has also been observed in the Vaccine Adverse Effect Reporting System (VAERS), the WHO Global Database of Individual Case Safety and vaccine clinical trial reports (Frontera et al., 2022; Harpaz et al., 2022; Rausch and Yue, 2022; Sadoff et al., 2022; Woodcock and Bartels, 2022).

To objectively evaluate the risks of COVID-19 vaccine adverse effects, they need to be compared to the risks associated with COVID-19 infections. There have been reports of tinnitus, vertigo, and sensorineural hearing loss occurring after COVID-19 infections (Jafari, Kolb and Mohajerani, 2022). However, a clear correlation and causation between these conditions and the virus have not been established (Fancello et al., 2021). Early studies noted cases of new-onset tinnitus in conjunction with hearing loss following COVID-19 infection (Beukes et al., 2020; Viola et al., 2021; Wichova, Miller and Derebery, 2021; Haider and Szczepek, 2022; Yellamsetty and Gonzalez, 2023). Subsequent case reports (Lamounier et al., 2020; Chirakkal et al., 2021; Daher et al., 2021; Yellamsetty, 2023) and comprehensive studies further indicated its frequent occurrence among affected patients (Kalcioglu, Cag, Kilic and Tuysuz, 2020; Munro, Uus, Almufarrij, Chaudhuri and Yioe, 2020). Some cases have been reported where patients experienced changes in hearing loss and perceived loudness (Khodaei, Safarabadi, Rad, Shakib and Nilechi, 2021; Haider and Szczepek, 2022; Yellamsetty, 2023).

We conducted a survey of people with self-reported tinnitus following COVID-19 vaccination and compared the survey responses with VAERS reports. The aims of the study were to characterize COVID-19 vaccination-related tinnitus, examine potential risk factors and explore effectiveness of treatments.

## Methods

### Survey

The study was approved by the University of Arizona Institutional Review Board. A cross-sectional survey was conducted from 12 August 2021, to 5 October 2021, among the Facebook group “Tinnitus and Hearing Loss/Impairment after COVID vaccination”, a support group that was founded for sufferers of tinnitus and hearing loss post COVID-19 vaccination. The online survey was announced in the Facebook group, and the survey link was posted. Participation in the survey was voluntary. Participants were pre-screened against inclusion criteria of vaccination status and the report of new or worsening tinnitus post COVID-19 vaccination. The participants provided consent by filling an online survey questionnaire that covered information on demography, general health before and after vaccination, noise exposure history, tinnitus assessment (Tinnitus Questionnaire, Tinnitus Functional Index and Visual Analogue Numeric Rating Scale), hearing evaluation (12-Question Short Form Speech, Spatial and Qualities of Hearing Scale) and attempted treatment of vaccination-related tinnitus (Hallam, Jakes and Hinchcliffe, 1988; Landgrebe et al., 2012; Noble, Jensen, Naylor, Bhullar and Akeroyd, 2013; Raj-Koziak et al., 2018). Survey responses included both clinical findings and self-assessments.

### Analysis of VAERS data

We conducted a retrospective review of VAERS reports, and COVID-19 vaccination data retrieved on 12/4/2021. All COVID-19 vaccine adverse effect reports were included, and classified by vaccine manufacturers, dose series, recipient sex, age, symptom/report onset and comorbidities. Only VAERS-coded/categorized symptoms were analyzed. VAERS analysis complements the survey study by providing information on a wider range of adverse events and following both COVID-19 and non-COVID-19 vaccinations. In addition, the large sample size of the VAERS data allows validation of some survey findings from a relatively small sample size. When comparing findings from VAERS and the survey data, only survey responses from the U.S. were included, because they were from the same vaccinated population.

### Statistical analysis

For comparison of non-normally distributed continuous variables, Mann–Whitney U or Kruskal–Wallis tests were used. Spearman correlation was used to measure the degree of association between two symptoms. Chi-squared tests were used to compare distributions of categorical variables. To determine if two events (**A** and **B**) were associated, we compared the frequency of observations where both events occurred (**F**_**A+B**_) and the expected frequency under the assumption of independence (i.e., **F**_**A**_ × **F**_**B**_) using a chi-squared test. The two events were considered associated if the chi-squared test rejected the independent assumption.

## Results

### Frequency of tinnitus by vaccine manufactures, lots and patient age groups

The Facebook group had approximately 2000 members at the time of the survey. We received responses from 414 members, and 398 of them completed the survey and met the inclusion criteria. These 398 respondents comprise 4 African-Americans, 2 American Indians, 19 Asians, 1 native Hawaiian/other pacific islander, 356 white/Caucasians and 16 of unspecified races. Thirty-two were of Hispanic, Latin or Spanish Origin. The respondents include 263 females, 122 males, 13 unreported. Of the 398 survey respondents, 223 took Pfizer/BioNtech, 120 Moderna, 28 AstraZeneca, 22 Johnson and Johnson, 1 Coronavac/Sinovac, 1 Covisheild, 1 Sputnik V, 1 mixed with Pfizer/BioNtech followed by Johnson and Johnson and 1 did not report (Table 1).

**TABLE 1:** Frequency and distribution of tinnitus cases across vaccine manufacturers.

	All survey cases	US survey cases	Total US doses administered*	VAERS tinnitus reports*	VAERS reports per million doses
Pfizer	224	167	273270797	6,488	23.74201734
Moderna	120	108	179679243	4,856	27.02593755
Janssen	22	21	16863349	1,175	69.6777372
AstraZeneca	28	2			
other	4	1			

*, All VAERS, and vaccine data were retrieved on 12/4/2021.

In addition to the survey, we also analyzed all VAERS cases that were reported between 1 January 2020 and 26 November 2021. The first COVID-19 vaccine-related tinnitus in the retrieved VAERS database was reported on 16 December 2020, and the last was reported on 26 November 2021. There were 750,273 cases in the database with unique VAERS_IDs, in which 668,978 cases reported adverse effects of COVID-19 vaccines. Tinnitus was reported in 12,957 of all the cases, with 12,532 tinnitus cases related to COVID-19 vaccines. The frequency of tinnitus was significantly higher for COVID-19 vaccines (12,532/668,978 = 1.87%) than for other vaccines (425/81,295 = 0.52%, *χ* ^2^ = 779.0002, *p* < 10^–5^), resulting in proportional reporting ratio of 3.58. The numbers of VAERS tinnitus reports per million doses administered were 23.7 for Pfizer, 27.0 for Moderna and 69.7 for Janssen. Since the Pfizer and Moderna vaccines require two doses, the number of tinnitus reports per million full vaccination is 47.4 and 54.0, respectively. The distribution of the US cases of tinnitus following COVID-19 vaccination amongst Pfizer, Moderna and Janssen vaccines are not different between our survey and the VAERS reports (*χ* ^2^ = 3.1972, *p* = 0.2022, See Table 1). Among COVID-19 vaccine-related tinnitus in the VAERS database, 8,422 cases reported 1,659 unique vaccine lot numbers (not individually verified). We found no evidence that the tinnitus cases were connected to specific lots of vaccines.

**FIGURE 1:**
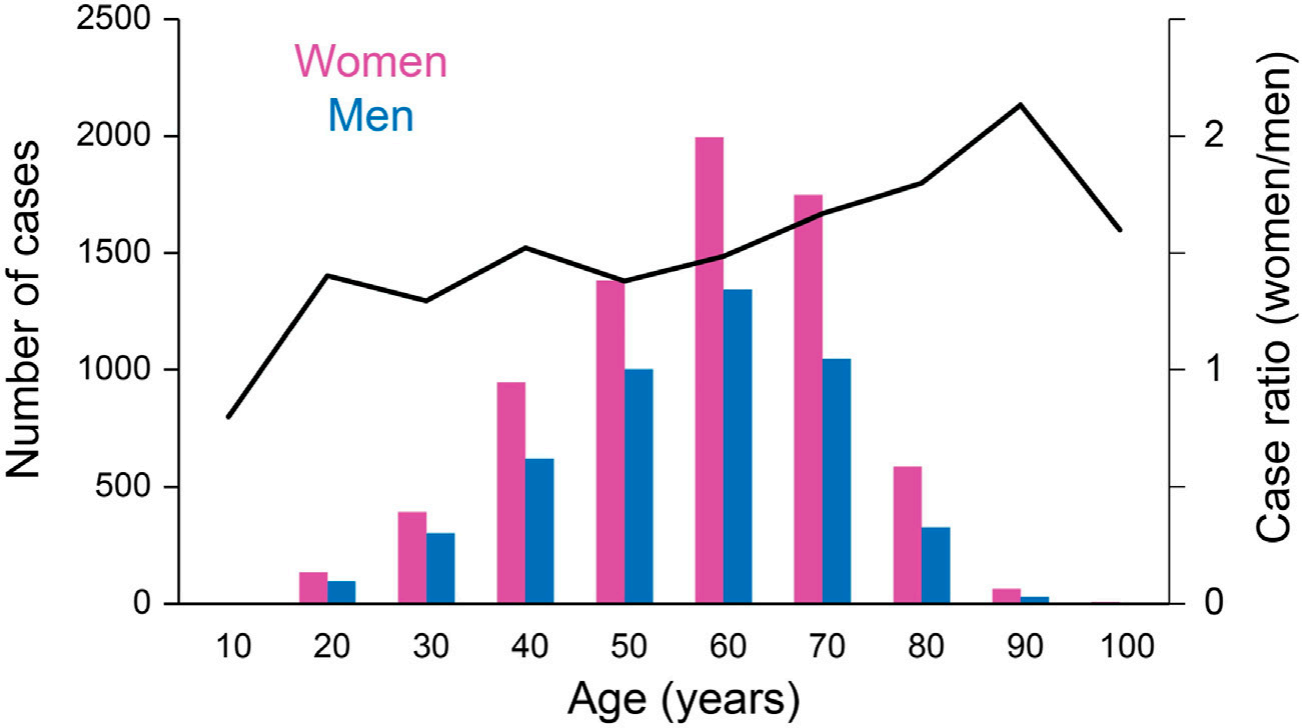
Sex differences across different age groups.

The VAERS cases were reported by people 6–92 years old, and a majority were in the age range of 40–70 years old (Figure 1). The number of tinnitus reports per million administered COVID-19 vaccines was higher for people between 18 and 64 years old compared to other age groups (Table 2; *χ* ^2^ = 907.5203, *p* < 10^–5^; vaccination data were available only for these age groups). A similar trend was also observed in the survey cases (Table 2; average age = 48.3 years old and SD = 13.0 years). COVID-19 vaccination-related tinnitus was reported more by women than men. In the 12,532 cases in the VAERS database, 4,903 were from males, 7,455 from females and 174 from people of unknown sex (Figure 1; *χ* ^2^ = 527.003, *p* < 10^–4^). In the survey responses, tinnitus was reported by 263 females, 122 males, and 13 unreported sex.

**TABLE 2:** Frequency of tinnitus per million administered vaccine doses by age groups.

Ages	Survey cases	Vaccine doses administered	VAERS tinnitus reports	VAERS reports/million doses
65-	42	124994116	2,726	21.81
18–64	333	310390205	9,605	30.94
12–17	0	28815487	178	6.18
5–11	0	6052786	17	2.81

### Dose series

In the 398 survey respondents, 98 (24.6%) had tinnitus before COVID-19 vaccination (Box 1). Significantly more cases reported **tinnitus symptoms** (hereafter defined as either the onset of tinnitus or worsening of preexisting tinnitus) following the first dose of COVID-19 vaccines than the second dose. For example, in the 98 cases with preexisting tinnitus, 69 (70.4%) had tinnitus symptoms worsened after the first dose of COVID-19 vaccines, and 29 (29.6%) after the second dose (*χ* ^2^ test against equal distribution, *χ* ^2^ = 16.33, *p* < 0.001). In the remaining 300 cases, 218 (72.7%) had tinnitus onset after the first dose of the vaccines, and 82 (27.3%) had tinnitus onset after the second dose (*χ* ^2^ test against equal distribution, *χ* ^2^ = 61.65, *p* < 0.001). Preexisting tinnitus did not influence case distribution between first and second doses (*χ* ^2^ test between cases with vs. without preexisting tinnitus, *χ* ^2^ = 0.1874, *p* = 0.6651). A breakdown of the dose series by vaccine manufacturers shows more tinnitus reports from the first than the second dose for two-dose vaccines from both Moderna and Pfizer (Table 3).

**TABLE 3:** Breakdown of the dose series by vaccine manufacturer in the survey responses.

Dose series	1st dose					2nd dose		Total
Pre-existing tinnitus	Pfizer	Moderna	Janssen	AZ	Other	Pfizer	Moderna	
Yes	41	16	7	5	0	20	9	98
No	120	56	15	23	4	43	39	300
Total	161	72	22	28	4	63	48	398

BOX 1 **Survey case breakdown.**

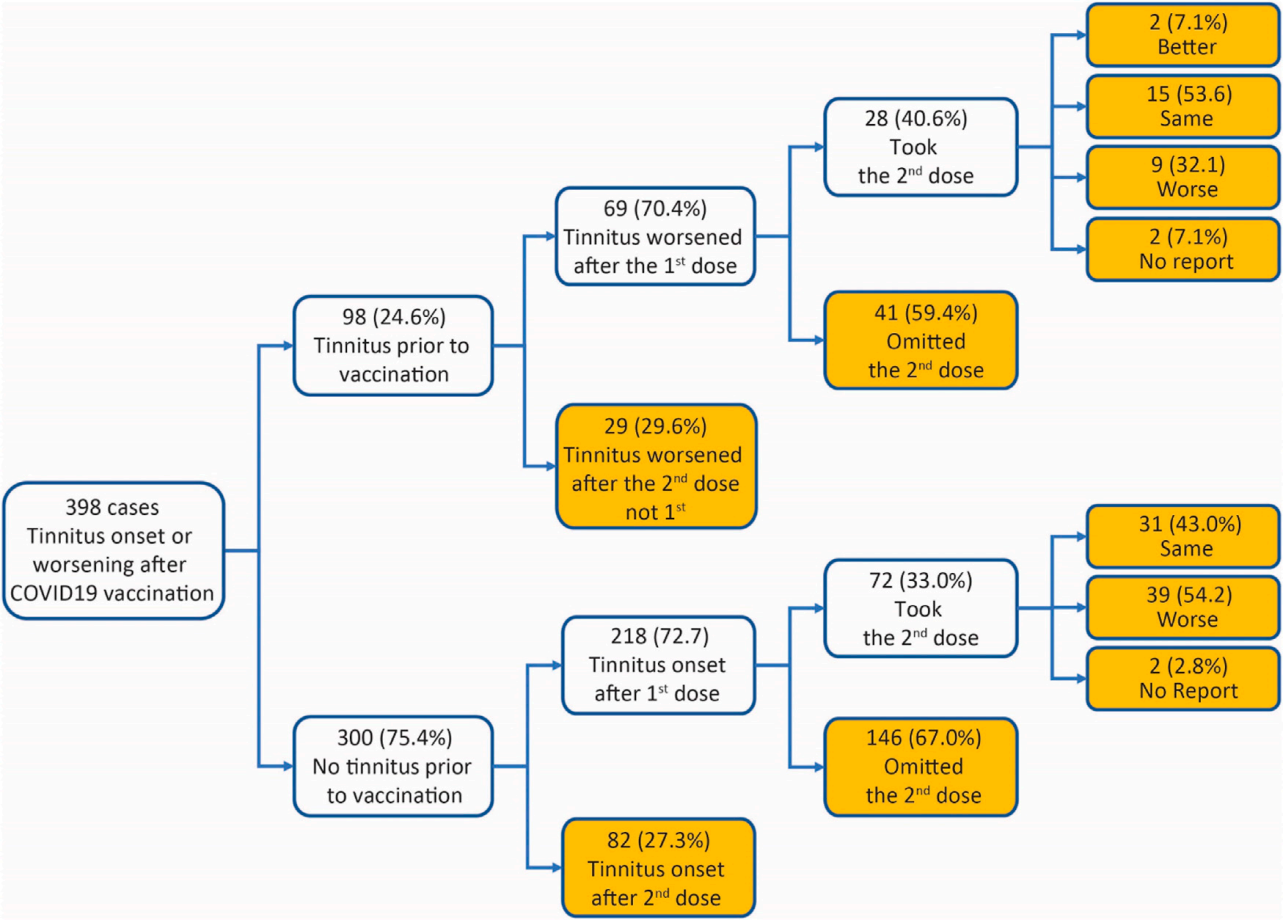

We estimated the number of tinnitus cases in VAERS reports per million first, second and third doses administered. For Pfizer and Moderna vaccines, we used the number of people fully vaccinated as the number of second doses administered. The number of first doses was calculated as the total doses administered minus second and booster doses. For Pfizer and Moderna vaccines, more tinnitus cases were reported per million of the first doses than the second doses (Table 4; Pfizer, *χ* ^2^ = 39.11, *p* < 10^–5^; Moderna, *χ* ^2^ = 44.91, *p* < 10^–5^). The number of second doses administered in Table 4 did not include the Pfizer/Moderna boosters that were administered to people who had received single-dose vaccines (i.e., Janssen or AstraZeneca).

**TABLE 4:** Breakdown of the dose series in the VAERS reports.

		Dose 1	Dose 2	Dose 3	Unknown	Total
Pfizer	Tinnitus cases	3,259	2,217	130	873	6,488
	Doses administered	137105789*	110769838**			
	Cases/million doses	23.77	20.01			
Moderna	Tinnitus cases	2,458	1,639	74	680	4,856
	Doses administered	87356154*	72099286**			
	Cases/million doses	28.14	22.73			
Janssen	Tinnitus cases	758	6	2	365	1,175
	Doses administered	15963295***				
	Cases/million doses	47.48				
total	Tinnitus cases	6,475	3,862	206	1918	12,519

*, Dose 1 = total doses administered—second doses - booster doses; **, # of fully vaccinated; ***, # of fully vaccinated.

Among the survey respondents who experienced tinnitus symptoms after the first dose of COVID-19 vaccines, 100 took the second dose. Worsening of tinnitus following the second dose was reported in 48 of the 100 cases (Box 1). The interval between the two vaccines was not different between those who reported the same level of tinnitus following the second dose and those who had worsening tinnitus (Same: mean 37.36 days, SEM 4.76 days, median 28 days, n = 44; worsening: mean 37.94 days, SEM 3.33 days, median 28 days, n = 47; Mann–Whitney test, *U* = 1,215, *p* = 0.145, two-tailed), suggesting no correlation between tinnitus worsening and timing of the second dose.

### Symptom onset

**FIGURE 2:**
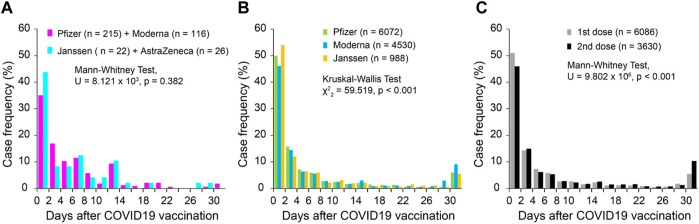
Onset latency of tinnitus following COVID-19 vaccination. **(A)** Survey cases; **(B)** VAERS cases grouped by vaccine manufacturers; and **(C)** VAERS cases grouped by dose series.

In the 398 survey cases, 384 reported symptom (tinnitus or worsening of existing tinnitus) onset latency relative to the vaccination date. Among all the cases, 36.8% had tinnitus onset within 2 days after the vaccination. The latency distribution was not different between mRNA vaccines (Pfizer and Moderna, n = 331) and non-mRNA vaccines (Janssen and AstraZeneca, n = 48; Mann Whitney test, *U* = 8.121 × 10^3^, *p* = 0.382, Figure 2A). In the VAERS database, 11,603 cases reported the symptom onset latency after COVID-19 vaccination, in which 5,665 cases (48.8%) had symptom onset within 2 days after vaccination. The onset latencies showed significant differences among Pfizer, Moderna and Janssen vaccines (Kruskal–Wallis Test, *χ* ^2^ = 59.519, *p* < 0.001, pairwise comparison, *p* < 0.005 for all pairs; Figure 2B). However, the differences were small and did not change the overall shape of the distributions. The tinnitus onset latency was slightly, but significantly, longer when it was associated with the second dose of COVID-19 vaccine compared to the first dose (Mann Whitney test, *U* = 9.802 × 10^6^, *p* < 0.001; Figure 2C).

### Tinnitus symptoms over time

**FIGURE 3:**
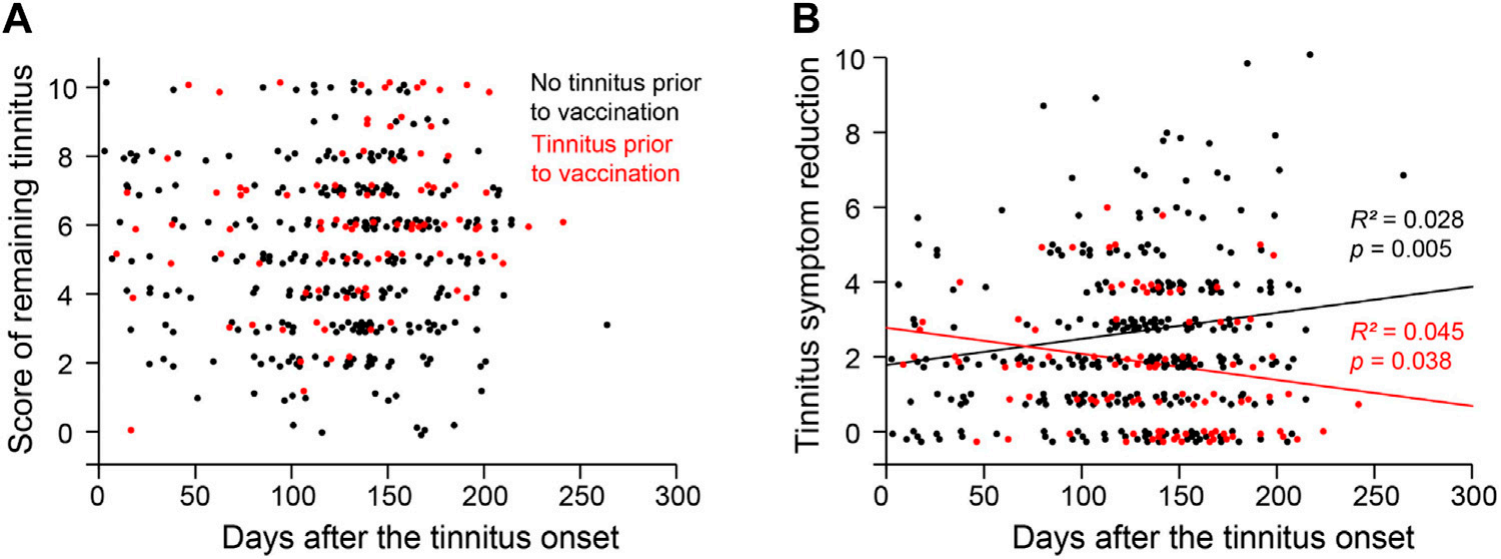
Distribution of Tinnitus severity over time. **(A)** The score of remaining tinnitus as a function of the duration/days from the tinnitus onset to the time of the survey. **(B)** The reduction of the tinnitus score from its peak level to the remaining level as a function of the duration/days from the tinnitus onset to the time of the survey.

The respondents of the survey reported their peak tinnitus levels and the remaining tinnitus levels on the day when they took the survey. The difference between the two levels is used as a measure of symptom improvement (i.e., worst tinnitus level minus remaining tinnitus level). We examined symptom improvement as a function of days from tinnitus onset. For respondents with no preexisting tinnitus, symptom improvement was positively correlated with days from tinnitus onset to the time of the survey (Figure 3; *R* ^2^ = 0.028, *p* = 0.005). For those with preexisting tinnitus, symptom improvement was negatively correlated with days between tinnitus onset and the time of the survey (*R* ^2^ = 0.045, *p* = 0.038). In both populations, passing time only explained a small portion of the variance.

### Treatment effects

Among the survey respondents, 187 took no medications, 139 took one medication (including vitamins), and 32 took more than one medication. We analyzed the effects of the medications on the level of peak tinnitus symptoms when it was the worst, the level of remaining tinnitus at the time of the survey, and the difference between the two levels as a measure of tinnitus improvement (Table 5). The level of remaining tinnitus was not different between those who took medication and those who did not. The worst level of tinnitus was greater for those who took one or more medications as compared to those who did not. Among the 139 respondents who took a single medication, 56.8% took prednisone. Compared to individuals who did not take medications, those took prednisone had significantly greater improvement in the reported level of tinnitus. No significant effects were observed with other medications.

**TABLE 5:** Effects of medications on tinnitus symptoms.

Group	Case number	Age	Remaining tinnitus level (0–10)	*p*-value	Peak tinnitus level (0–10)	*p*-value	Tinnitus improvement (0–10)	*p*-value
		Median (IQR)	Mean ± SD	^a^	Mean ± SD	^a^	Mean ± SD	^a^
			Median (IQR)		Median (IQR)		Median (IQR)	
No medication	187	48.3 ± 13.6	5.25 ± 2.29		7.41 ± 1.89		2.16 ± 1.62	
		47.8 (22.8)	5 (3)		7 (3)		2 (2)	
Multiple medications	32	46.5 ± 11.7	5.06 ± 3.03	0.743	8.04 ± 1.66	0.004^##^	3.34 ± 2.84	0.021^#^
		44.3 (19.4)	5 (5.75)		9 (3)		3.5 (4)	
Single medication	139	48.8 ± 12.6	5.42 ± 2.38	0.507	8.06 ± 1.85	0.001^##^	2.63 ± 2.09	0.049^#^
		49.4 (19.9)	6 (4)		8 (3)		2 (3)	
Prednisone	79	47.4 ± 13.3	5.54 ± 2.30	0.151	8.24 ± 1.75	0.000^###^	2.70 ± 2.07	0.040^#^
		47.5 (20.0)	6 (3)		8 (3)		2 (3)	
Other steroids	31	50.3 ± 11.3	5.29 ± 2.36	0.446	7.84 ± 1.93	0.077	2.55 ± 1.89	0.414
		49.8 (13.5)	6 (4)		8 (3)		2 (3)	
Antihistamines	12	53.2 ± 8.8	4.83 ± 1.83	0.272	8.00 ± 1.59	0.159	3.17 ± 2.60	0.186
		55.0 (11.0)	5 (3)		8 (3.5)		2 (5)	
NSAIDs	10	45.1 ± 11.2	6.30 ± 3.09	0.104	8.00 ± 2.49	0.089	1.70 ± 1.77	0.187
		44.2 (16.3)	7.5 (5.75)		8.5 (3)		2 (2.5)	
Vitamins	7	55.9 ± 13.6	4.43 ± 2.82	0.108	7.14 ± 2.04	0.343	2.71 ± 2.21	0.486
		60.2 (27.3)	4 (4)		7 (3)		3 (4)	

^a^ A Mann-Whitney U test on differences in tinnitus levels between No medication group and other individual groups. #, *p* < 0.05; ##, *p* < 0.005; ###, *p* < 0.001. Not all survey participants responded to this inquiry.

**FIGURE 4:**
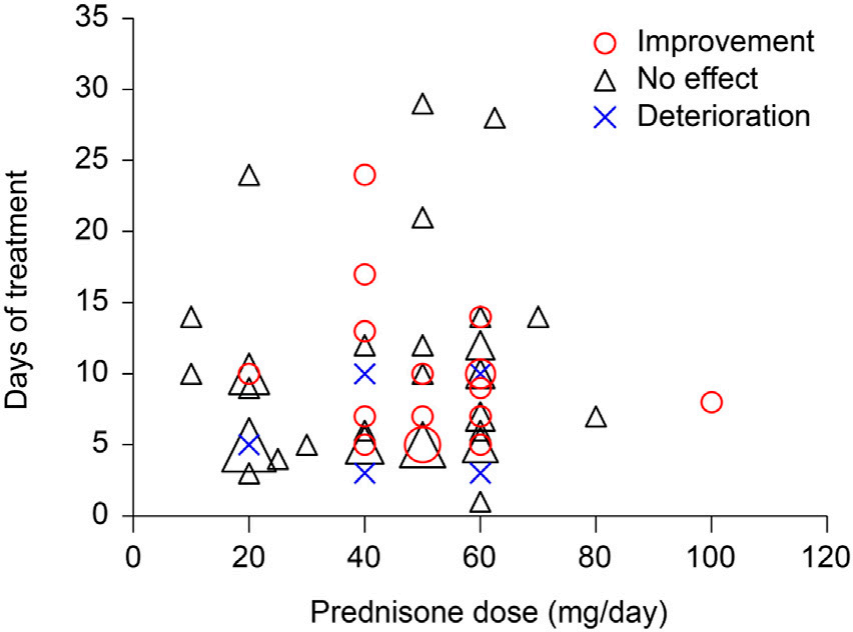
Effects of prednisone as a function of drug dose and treatment duration.

Among the 90 survey respondents who took prednisone (79 took prednisone only and 11 took multiple medications), 56 reported no effects, 28 reported improvement, and 6 reported worsening symptoms, all by self-assessment. Seventy respondents reported both prednisone doses and treatment durations (Figure 4). Prednisone below 40 mg/day appears to be ineffective, with only 1 in 20 reporting symptom improvement and 1 reporting symptom deterioration. By contrast, at doses of 40 mg/day or higher, 17 of the 50 respondents reported improvement in tinnitus symptoms and only 4 reported worsening of tinnitus. These results indicate a dose dependence of the effect of prednisone on tinnitus symptoms following COVID-19 vaccines (*χ* ^2^ = 7.0309, *p* = 0.0297). This statistically significant dose dependence suggests that the improvement of tinnitus symptoms following prednisone treatment is an effect of prednisone and not spontaneous recovery or a placebo effect.

Ninety-two of the 187 respondents who did not take medications received alternative treatment including acupuncture, massage and sound. None of the treatments led to significant improvement in the level of tinnitus compared to the untreated group. Only eight respondents received acupuncture and their tinnitus improvement scores were trending higher than the untreated group (Table 6; *p* = 0.016).

**TABLE 6:** Effects of alternative treatments.

Group	Case number	Age	Tinnitus current level (0–10)	*p*-value	Tinnitus worst level (0–10)	*p*-value	Tinnitus improvement (0–10)	*p*-value
		Median (IQR)	Mean ± SD	^**^	Mean ± SD	^**^	Mean ± SD	^***^
			Median (IQR)		Median (IQR)		Median (IQR)	
Untreated	92	48.0 (23.3)	5.28 ± 2.42		7.27 ± 2.03		1.99 ± 1.57	
			5.5 (4)		7 (3)		2 (2)	
Multiple treatments	42	47.9 (22.2)	5.40 ± 2.34	0.905	7.83 ± 1.92	0.152	2.43 ± 1.80	0.101
			5 (2.25)		7.5 (3.25)		2 (3)	
Single treatments	53	47.8 (23.0)	5.11 ± 2.12	0.680	7.47 ± 1.59	0.719	2.36 ± 1.70	0.105
			5 (3)		8 (2)		2 (3)	
Acupuncture	8	51.7 (21.9)	4 ± 1.77	0.126	6.63 ± 1.41	0.304	2.63 ± 1.19	0.106
			3 (2.75)		6.5 (2)		2.5 (1)	
Massage	10	48.8 (24.2)	6.00 ± 2.58	0.479	8.10 ± 1.29	0.226	2.1 ± 1.66	0.416
			5.50 (3.75)		8.00 (2.25)		2.00 (2.5)	
Music/Sound Therapy	32	44.8 (23.8)	5.31 ± 1.94	0.831	7.44 ± 1.64	0.837	2.13 ± 1.66	0.345
			6 (3)		7 (2.75)		2 (2.75)	

**A Mann-Whitney U test was run to determine if there were differences in tinnitus levels between untreated and other individual monotherapy groups. ***One-tailed test was used to determine if there were differences in tinnitus improvement between untreated and other individual monotherapy groups.

### Comorbidities

VAERS database retrieved on 4 December 2021, had 699,839 COVID-19 vaccine-related reports. The 10 most common complaints are headache (120,205 reports), pyrexia (101,523), fatigue (99,947), chills (87,576), pain (85,942), dizziness (68,904), nausea (68,079), pain in extremity (64,879), myalgia (41,430), arthralgia (40,222). Tinnitus (12,532) is the 44th complaint. In reports with tinnitus complaints, the 10 most common comorbidities are headache, dizziness, fatigue, nausea, pyrexia, chills, pain, ear discomfort, pain in extremity and vertigo (Table 7). In COVID-19 vaccine-related tinnitus reports, there were significantly more complaints of ear discomfort, vertigo, dizziness, and headache than would be expected if the symptoms were independent of tinnitus (Table 7), suggesting that these symptoms are positively correlated with tinnitus. Interestingly, there were significantly fewer complaints of pyrexia, pain, chills, pain in extremity and fatigue than would be expected, indicating that they are negatively correlated to tinnitus (Table 7).

**TABLE 7:** Ten most common complaints in VAERS COVID-19-related tinnitus reports.

Symptom	Total # of cases *	Expected # of cases with tinnitus	Reported # of case with tinnitus **	Observed/expectation ratio	*p*-value of *χ* ^2^ tests
Headache	120,205	2,153	2,307	1.07	0.000289
Dizziness	68,904	1,234	2018	1.64	<0.00001
Fatigue	99,947	1790	1,589	0.89	<0.00001
Nausea	68,079	1,219	1,174	0.96	0.1778
Pyrexia	101,523	1818	1,095	0.60	<0.00001
Chills	87,576	1,568	1,005	0.64	<0.00001
Pain	85,942	1,539	960	0.62	<0.00001
Ear discomfort	2,423	43	850	19.59	<0.00001
Pain in extremity	64,879	1,162	786	0.68	<0.00000
Vertigo	7,297	131	740	5.66	<0.00001

*, total number of COVID-19, vaccine-related VAERS, cases reported by 4 December 2021, is 699,839; **, total number of COVID-19 vaccine-related tinnitus reports in the VAERS, database is 12,532.

As tinnitus is believed to be a symptom of neurological conditions with psychiatric consequences, we examined neurological and psychiatric symptoms that were not among the top 10 comorbidities (Table 8). We found that visual impairments, Meniere’s disease, migraine, confusional state, memory impairment, anxiety, and depression were all positively correlated with COVID-19 vaccination-related tinnitus.

**TABLE 8:** Other neurological and psychiatric symptoms in VAERS COVID-19-related tinnitus reports.

Symptom	Total # of cases *	Expected # of cases with tinnitus	Reported # of case with tinnitus **	Observed/expectation ratio	*p*-value of *χ* ^2^ tests
Vision blurred	6,623	118	341	2.88	<0.00001
Visual impairment	3,701	66	227	3.43	<0.00001
Movement disorder	1,581	28	20	0.71	0.101916
Meniere’s disease	78	1.34	22	15.75	<0.00001
Migraine	8,507	152	262	1.72	<0.00001
Confusional state	5,147	92	134	1.45	0.00004
Memory impairment	1,578	28	59	2.09	<0.00001
Anxiety	7,681	138	262	1.90	<0.00001
Depression	1,071	19	62	3.23	<0.00000

*, total number of COVID-19, vaccine-related VAERS, cases reported by 4 December 2021, is 699,839; **, total number of COVID-19 vaccine-related tinnitus reports in the VAERS, database is 12,532.

The VAERS reports have 31 categories of symptoms that are related to hearing loss, deafness and other ear-related complaints. We listed several of these symptoms in Table 9. The frequency of tinnitus complains was significantly higher among those who had one or more of these 31 symptoms (2,409 out of 9,325 cases, 25.83%) than those who did not have any of the symptoms (10,124 out of 692,218, 1.47%; *χ* ^2^ = 18,391.78, *p* < .00001). For example, tinnitus was reported in 48% of the cases with any deafness symptoms following COVID-19 vaccination, and in 35% of the reports with sudden hearing loss. However, it should be noted that only 19.22% (2,409 out of 12,532) of the tinnitus cases registered any hearing- and ear-related complaints, and only 10.22% of cases (1,281 out of 12,532) reported hearing loss/deafness. By contrast, approximately 60% of people with tinnitus in the general population also showed hearing loss (Batts and Stankovic, 2024). This difference suggests that COVID-19 vaccination-related tinnitus and tinnitus in the general population likely have different etiologies.

**TABLE 9:** Common hearing- and ear-related symptoms in VAERS reports.

Symptoms*	# Of cases in all COVID-19 vaccine reports	Expected # of cases in vaccine tinnitus reports	# Of case in vaccine tinnitus reports	Observed/expectation ratio	*p*-value of *χ* ^2^ tests
Deafness	1,343	24.0	652	27.11	<0.00001
Ear pain	3,564	63.8	492	7.71	<0.00001
Deafness unilateral	971	17.4	465	26.74	<0.00001
Deafness neurosensory	282	5.0	134	26.53	<0.00001
Ear congestion	274	4.9	95	19.36	<0.00001
Sudden hearing loss	272	4.9	95	19.50	<0.00001
Ear infection	414	7.4	63	8.50	<0.00001
Deafness bilateral	126	2.3	60	26.59	<0.00001
Middle ear effusion	175	3.1	51	16.27	<0.00001
Ear disorder	131	2.3	25	10.66	<0.00001

*, many of the symptoms were over-lapping and a report could have complaints of multiple symptoms.

There were 605 complaints of hyperacusis in all COVID-19 vaccine-related VAERS reports, with 177 of them comorbid with tinnitus. The frequency of hyperacusis was significantly higher among those with tinnitus symptoms (i.e., hyperacusis occurred in 0.086% of all COVID-19-related reports, and 1.412% of COVID-19-related tinnitus reports; *χ* ^2^ = 1973.76, *p* < .00001; proportional reporting ratio, 16.42).

**FIGURE 5:**
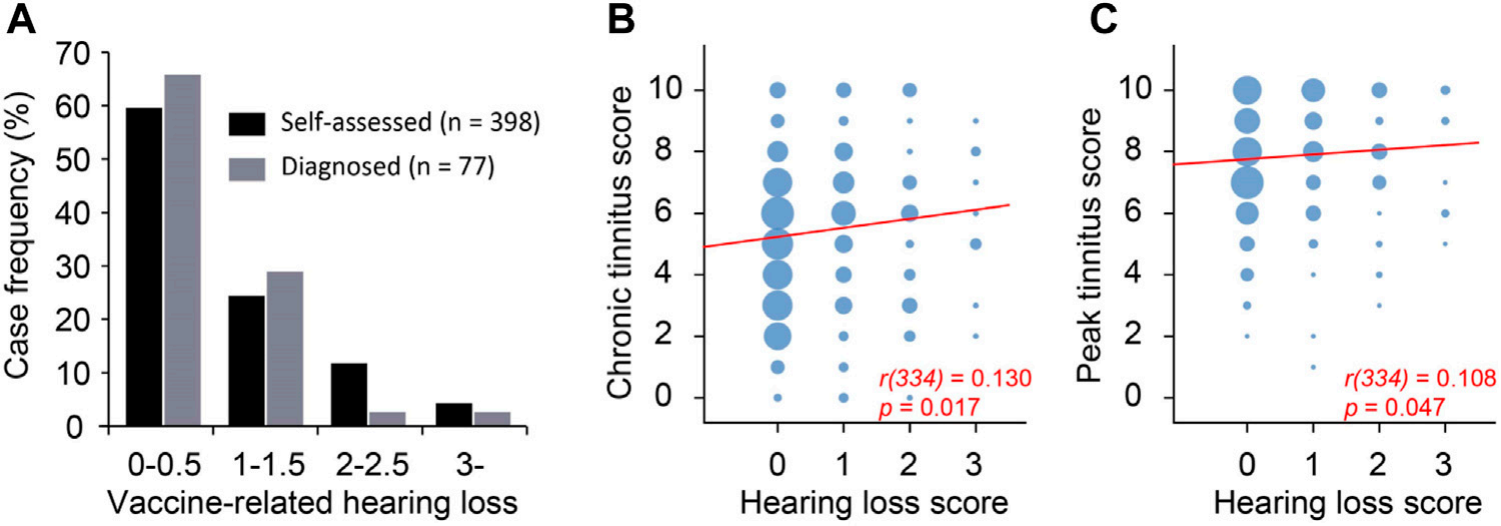
Peak tinnitus level was weakly correlated with hearing loss. **(A)** Self-assessed and diagnosed hearing loss have similar distribution. **(B)** Chronic tinnitus as a function of hearing loss. **(C)** Peak tinnitus as a function of hearing loss. Area of the bubble represents number of cases. Spearman correlation.

We asked the survey respondents to report **self-assessed** hearing loss on a scale of 0–3 (no-0, mild-1, moderate-2, severe-3) that they believed to be related to the vaccination. In addition, 128 respondents had audiological tests before COVID-19 vaccination, 280 had tests after the vaccination, and 77 reported levels of hearing loss from the two tests (i.e., before and after vaccination) on the same scale of 0–4 (no-0, mild-1, moderate-2, severe-3, profound-4). Of the 280 respondents who took audiological tests after vaccination, 29 reported hearing loss in the left ear, 21 in the right ear, and 125 in both ears. The remaining 105 respondents reported normal hearing in both ears. The hearing loss in some of the 175 cases presumably existed before the vaccination and were not associated with the vaccination. To estimate the worsening of hearing following COVID-19 vaccination, the difference between biaural averages of the test-measured hearing loss after vs before the vaccination was calculated as **diagnosed** vaccination-associated hearing loss (Figure 5A). Approximately 66% of the 77 respondents showed either no worsening of hearing in any ear or mild worsening in only one ear (i.e., average biaural hearing loss rating ≤0.5). For these 77 respondents, the self-assessed hearing loss and diagnosed hearing loss was significantly correlated (*R* ^2^ = 0.695, *p* < 0.001). The self-assessed and diagnosed hearing loss had similar distributions (Figure 5A), validating self-assessed hearing loss as a useful measure.

A total of 336 survey respondents took the survey more than 60 days after tinnitus onset. The tinnitus they experienced at the time of survey was considered residual/chronic. The scores of the chronic and peak tinnitus were significantly correlated with self-assessed hearing loss (Figure 5, B-C). However, the correlation was rather weak as indicated by the low Spearman’s rho values (Figure 5, B-C). This is likely because many respondents reported tinnitus without COVID-19 vaccination-associated hearing loss (Figure 5A).

**FIGURE 6:**
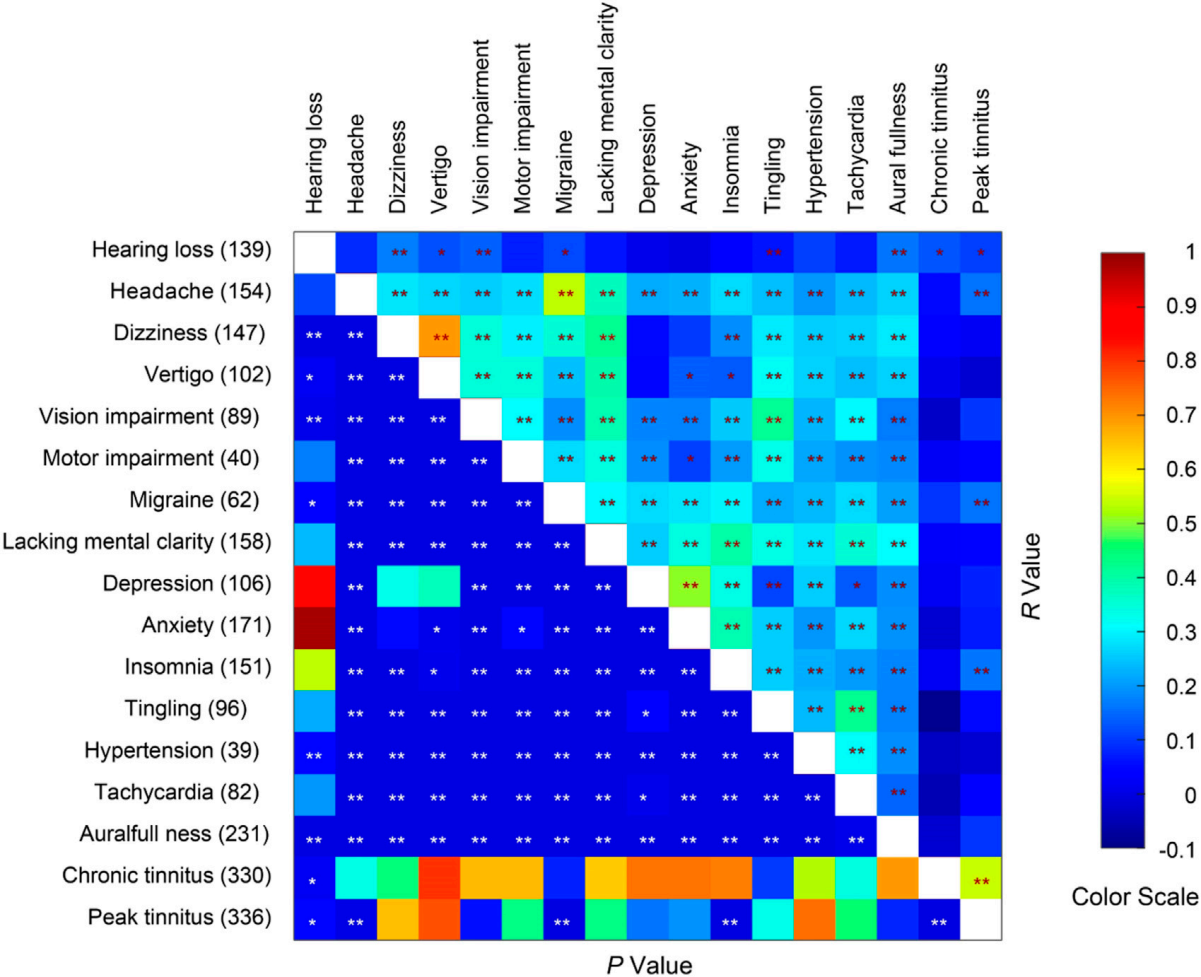
Spearman correlations COVID-19 vaccination-related symptoms. The number of respondents reporting each medical condition is given on the left axis. * and ** indicate *p* < 0.05 and *p* < 0.01 respectively.

Many of the survey respondents reported multiple neurological, psychiatric and other physical symptoms in addition to tinnitus. We asked the respondents to rate their symptoms on a scale of 0–3. A pair-wise correlation analysis on some common symptoms revealed that chronic tinnitus scores were only correlated with hearing loss, whereas peak tinnitus scores were correlated with the degrees of headache (Figure 6; n = 336, *r* = 0.159, *p* = 0.004), migraine (*r* = 0.162, *p* = 0.003), and insomnia (*r* = 0.163, *p* = 0.003). Peak tinnitus scores were also correlated with chronic tinnitus scores (*r* = 0.548, *p* < 9.84 × 10^−28^).

As many of the symptoms are pairwise correlated, we performed multiple linear regression with tinnitus scores as dependent variables and other symptoms as predictors to explore potential connections between tinnitus scores and scores of other symptoms. Overall, the 15 non-tinnitus symptoms predicted peak tinnitus scores better (*R* ^2^ = 0.089, *F*_15,335_ = 2.091, *p* = 0.10) than did the chronic tinnitus score (*R* ^2^ = 0.053, *F*_15,335_ = 1.204, *p* = 0.267). Hearing loss was a significant predictor of the chronic tinnitus score, and insomnia and hypertension were significant predictors of the peak tinnitus score (Table 10). Interestingly, COVID-19-related hypertension was negatively correlated with the peak tinnitus score (*B* = −0.369).

**TABLE 10:** Comorbidities as predictors of chronic and peak tinnitus levels.

Symptoms	Chronic tinnitus				Peak tinnitus			
	B	Std. Error	Beta	P	B	Std. Error	Beta	P
(Constant)	5.072	0.257		0.000	7.333	0.191		0.000
HL*	0.367	0.163	0.127	**0.025**	0.185	0.121	0.085	0.126
Headache	0.163	0.158	0.073	0.302	0.120	0.117	0.071	0.307
Dizziness	0.209	0.200	0.091	0.299	−0.156	0.149	−0.091	0.293
Vertigo	−0.143	0.200	−0.060	0.477	−0.101	0.149	−0.056	0.495
Vision	−0.103	0.217	−0.031	0.634	0.241	0.161	0.095	0.136
Motor	0.059	0.273	0.013	0.831	−0.033	0.203	−0.010	0.869
Migraine	0.113	0.209	0.038	0.591	0.228	0.155	0.102	0.143
Brain fog	0.218	0.174	0.092	0.210	−0.118	0.129	−0.065	0.361
Depression	−0.028	0.165	−0.011	0.866	0.027	0.122	0.015	0.825
Anxiety	−0.033	0.140	−0.016	0.817	−0.033	0.104	−0.022	0.750
Insomnia**	0.095	0.137	0.045	0.487	0.288	0.101	0.178	**0.005**
Tingling	−0.199	0.183	−0.073	0.279	0.050	0.136	0.025	0.712
Hypertension**	−0.300	0.246	−0.076	0.223	−0.369	0.182	−0.123	**0.044**
Tachycardia	−0.197	0.184	−0.069	0.286	0.026	0.136	0.012	0.848
Aural fullness	−0.156	0.129	−0.072	0.229	0.095	0.096	0.058	0.321

* indicates significant predictor of chronic tinnitus level, ** denotes significant predictor of peak tinnitus level, and bold font shows *p* < 0.05.

### Risk factors

**FIGURE 7:**
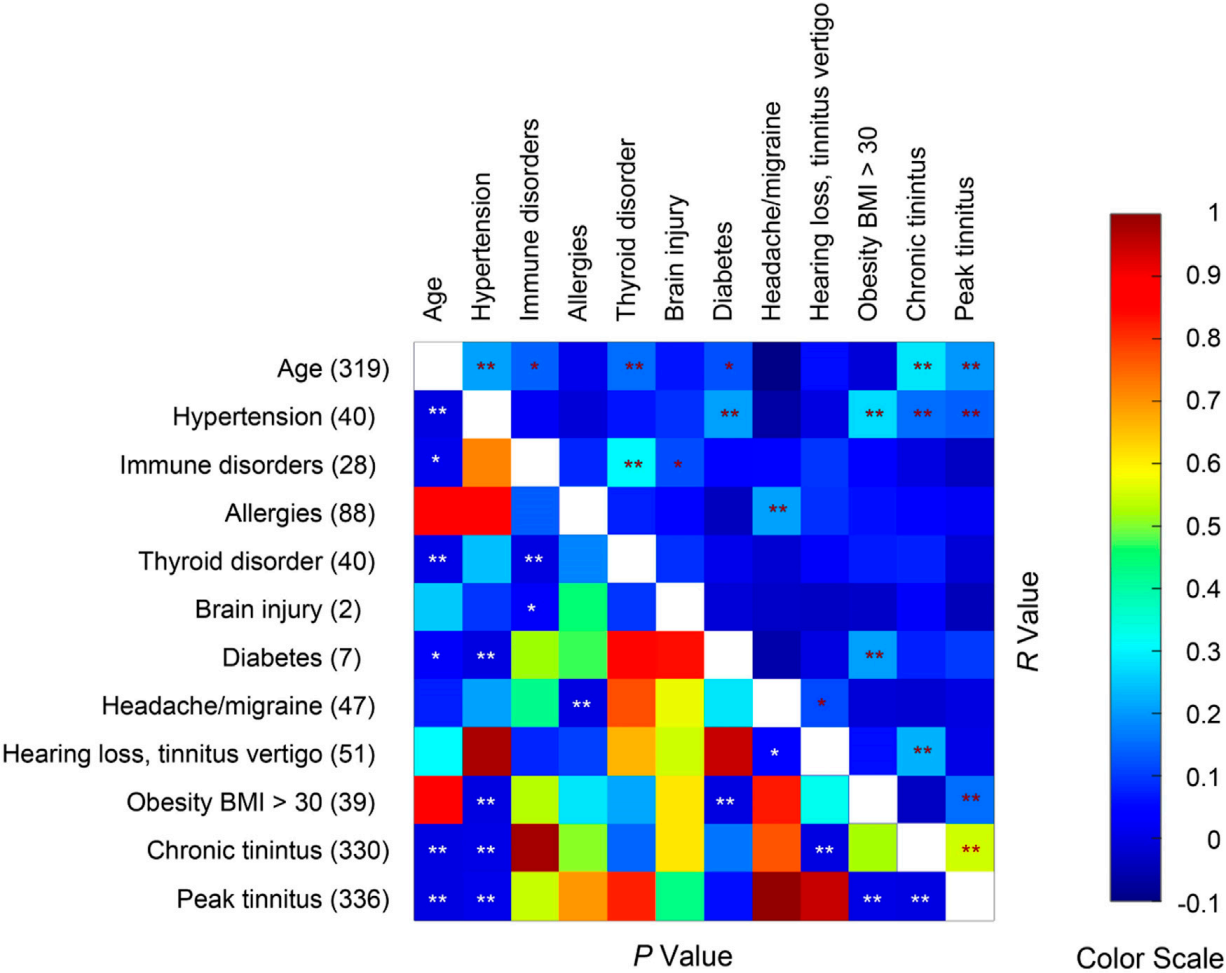
Spearman correlations between age, preexisting medical conditions, and post-vaccine tinnitus severities. Sample size = 336. Seventeen respondents did not report age. The number of respondents reporting each medical condition is given on the left axis. * and ** indicate *p* < 0.05 and *p* < 0.01 respectively.

To evaluate what medical conditions may exacerbate tinnitus following COVID-19 vaccination, we asked the survey respondents whether they had one of more of 11 medical conditions. Age was also considered a preexisting condition. None of the respondents reported stroke or multiple sclerosis. We examined the correlations between the remaining 10 preexisting medical/age conditions with chronic tinnitus score and peak tinnitus score (Figure 7). The chronic tinnitus score was significantly correlated with age, hypertension and hearing/tinnitus/vertigo. The peak tinnitus score was significantly correlated with age, hypertension and obesity. There was a trending correlation between diabetes and the peak tinnitus score (*p* = 0.061). Many of the preexisting conditions were correlated themselves. For example, hypertension, diabetes and obesity were correlated, possibly indicating metabolic syndrome. Immune disorders (immune deficiency and autoimmune disease) and thyroid diseases are also correlated. The survey results suggest that metabolic syndrome is a risk factor for severity of post-vaccination tinnitus, but immune disorder is not.

### Genetic and lifestyle influences

Given the low VAERS report frequency of tinnitus following COVID-19 vaccination (i.e., 47–70 cases/million), the expected frequency that two members of the same families independently develop COVID-19 vaccination-related tinnitus is low. For example, for an extended family of 50 members, if one member develops COVID-19 vaccination-related tinnitus, the likelihood that at least one more member *independently* develops COVID-19 vaccination-related tinnitus is no more than 0.35%. For a household of 3 people (US average 2.50 ± 0.01, 2022, United States Census Bureau), this number drops to 0.014%. By contrast, 36 of the 398 survey respondents from 26 households reported that at least another household member also developed COVID-19 vaccination-related tinnitus. Among them, 3 members from 2 households each, 2 members from 6 households and 1 member from 18 households participated in the survey. In the 26 households, 7 had at least 2 genetically related individuals showing COVID-19 vaccination-related tinnitus, and 19 had at least 2 genetically unrelated individuals developing the symptoms. In addition to the household cases, 17 respondents reported that their genetic relatives who lived in a different household also developed COVID-19 vaccination-related tinnitus, but they did not participate in the survey. The frequencies of both the genetic/non-household cases (17/398 = 8.58%) and non-genetic/household cases (19/398 = 9.59%) in the survey response far exceeded our upper limit estimates under the independent assumption (0.35% and 0.014%, χ2 test, *p* < 10^–4^). Thus, unless the VAERS analysis vastly underestimated COVID-19 vaccination-related tinnitus, the genetic and house concordance in the survey results suggest that both genetics and household lifestyle influence the risk of COVID-19 vaccination-related tinnitus.

## Discussion

### Limitations

In the present study, we analyzed data from a survey of people who developed (or exacerbated existing) tinnitus following COVID-19 vaccination, and from VAERS cases reported between 1 January 2020 and 26 November 2021. It should be noted that neither of the two sets of data were from randomly sampled populations. The survey cases probably were biased for people with more severe symptoms because people with moderate/resolving symptoms could be less motivated to participate in the survey. The VAERS database are likely incomplete since problems in case entry had been reported (Block, 2023). To mitigate some of these problems, we focused on comparisons within each database (e.g., tinnitus cases after the first vs. second doses). When it was possible, we also cross-validated findings in the survey and the VAERS databases (e.g., more cases following the first dose, rapid onset, sex ratio … ). The remaining limitations need to be addressed in future studies of randomly sampled populations with proper control groups.

### COVID-19 vaccination increases the risk for tinnitus

The main question we seek to answer is whether the reported tinnitus following COVID-19 vaccination was related to the vaccination event. Several findings in the present study suggest that COVID-19 vaccine increases the risk of tinnitus. First, the frequency of tinnitus reports in the VAERS database is higher for COVID-19 than for other vaccines. Second, the frequency of tinnitus reports was higher for the first than the second dose for the 2-dose vaccines, which would not be expected if the tinnitus and the vaccination were independent. Third, for the 2-dose vaccines, the survey respondents who developed tinnitus after the first does had 50% chance of worsening tinnitus symptoms after the second dose, which is much higher than the frequency of tinnitus for the general population. Fourth, the tinnitus onset was sharply time-locked to the vaccination, with nearly half of the cases occurring within 2 days of the vaccination. Fifth, tinnitus occurring after COVID-19 vaccination was associated with seemingly unrelated neurological disorders such as vision impairment, confusional state and memory impairment. The cooccurrence of these symptoms suggest that COVID-19 vaccines had a broader impact on the nervous system in the susceptible individuals.

### Risk factors for COVID-19 vaccination-related tinnitus

There were approximately 47–70 VAERS tinnitus reports per million fully vaccinated population. What makes those people susceptible to COVID-19 vaccination-related tinnitus whereas a great majority of the vaccinated population were not affected? Findings in the present study suggest that interactions between COVID-19 vaccination and pre-existing risk factors (i.e., medical/health conditions) lead to the reported tinnitus. For example, the higher number of tinnitus cases following the first than the second dose is consistent with the hypothesis. If a majority of the people with the hypothetic risk factors developed tinnitus following the first dose, much fewer of the remaining vaccine recipients (those did not develop tinnitus after the first dose) would have the risk factor. Those who developed tinnitus following the first dose of vaccines likely had the risk factors, and therefore had higher chances of worsening tinnitus symptoms following the second dose compared to the first dose. Those who did not develop tinnitus following the first dose likely did not have the risk factors, and therefore had lower incidence of tinnitus (compared to the general population) following the second dose.

Although our survey study did not exhaustively examine risk factors of COVID-19 vaccination-related tinnitus, it did provide some interesting insights. We found that age, preexisting hypertension and obesity were significantly correlated with the severity of COVID-19 vaccination-related tinnitus. Diabetes (with only 6 cases) was also trending correlated with the tinnitus severity. Hypertension (Chen et al., 2021; Du et al., 2021; Li et al., 2021; Tan et al., 2021), obesity (Agarwal et al., 2021; Amin et al., 2021; Demeulemeester et al., 2021; Sattar and Valabhji, 2021) and diabetes (Berrajaa et al., 2021; Li et al., 2021; Sathish, 2021) are major risk factors for severity and mortality in COVID-19 patients. The common risk factors for the COVID-19 and COVID-19 vaccination-related tinnitus suggest that they might share some common mechanisms. Tinnitus is generally associated with hypertension, diabetes and obesity (Gibrin, Melo and Marchiori, 2013; Yang, Ma, Zheng, Yang and Lin, 2015; Figueiredo, Azevedo and Penido, 2016; Mousavi, Sajadinejad, Khorsandi and Farhadi, 2021; Ameen et al., 2022; Patel et al., 2022; Ramatsoma and Patrick, 2022; Özbey-Yücel and Uçar, 2023). Further studies are needed to determine whether these metabolic disorders interact synergistically with COVID-19 vaccination.

Pre-vaccination psychological health as a risk factor has not been considered in our survey. Stress, anxiety and depression have been associated with risk of tinnitus (Patil, Alrashid, Eltabbakh and Fredericks, 2023). The social distancing-related stress and emotional impact could contribute to the risk of COVID-19 vaccination-related tinnitus.

### High-dose steroids as a treatment option

In the survey responses, we observed a dose-dependent effect of prednisone for treatment of COVID-19-related tinnitus. Seventeen of 50 survey respondents who received prednisone at or higher than a dose of 40 mg/day showed improvement in tinnitus symptoms by self-assessment and 4 showed deterioration. We do not have information on the delays between tinnitus onset and the start of the medication. It is possible that long delays weakened the drug treatment effect in some respondents. It is also possible that the heterogeneity of the tinnitus pathologies among the respondents led to the varied treatment outcomes.

### Mechanisms of COVID-19 vaccination-related tinnitus

Immune response to SARS-CoV-2 viruses could lead to cytokine storms resulting in severe injury to the body systems. COVID-19 vaccines were designed to induce body immune responses. If unregulated, overactive immune responses to the vaccines could lead to neuroinflammatory response and tinnitus. However, this hypothesis of overactive immune response is not supported by our findings. The systemic immune response develops gradually over many days after immunization, whereas the COVID-19 vaccination-related tinnitus developed rapidly, often within 2 days. Furthermore, body immune response is stronger following the second than the first dose of the vaccines. However, more tinnitus cases were reported following the first than the second dose. These mismatches suggest that the reported tinnitus was not due to overactive systemic immune responses to the vaccines. Instead, it is likely the vaccine adverse effect was mediated by a more direct pathway. Spike proteins have been shown to disrupt the blood-brain barrier (DeOre, Tran, Andrews, Ramirez and Galie, 2021; Rhea et al., 2021; Zhang et al., 2021; Petrovszki et al., 2022), activate microglia/neuroinflammation (Frank et al., 2022; Albornoz et al., 2023) and cause neuronal death (Oh et al., 2022). It may also cause aggregation of β-amyloid proteins, prions, α-synuclein and tau, potentially leading to neurodegeneration (Tavassoly, Safavi and Tavassoly, 2020; Idrees & Kumar, 2021; Chakrabarti et al., 2022). It remains to be determined whether COVID-19 vaccines, which are modified forms of the spike protein, can disrupt brain function in similar ways and cause tinnitus and other neurological symptoms. In addition to direct interaction with the brain cells, COVID-19 vaccines can cause rapid systemic inflammatory responses secondary to reactogenicity of the vaccines (Heo et al., 2022). COVID-19 vaccines have also been associated with endothelial dysfunction and vascular inflammation (Terentes-Printzios et al., 2022; Yoshimoto et al., 2023; Cernera et al., 2024), including brain blood vessels (Ariyoshi et al., 2023). These systemic and vascular inflammation could disrupt blood-brain barrier and other brain function, and potentially lead to tinnitus and other mental health issues (G. Yang, Parkhurst, Hayes and Gan, 2013).

Several other mechanisms may contribute to COVID-19 vaccination-related tinnitus. For example, the human inner ear expresses angiotensin-converting enzyme-2 (ACE2) receptor for the spike protein (Jeong et al., 2021). If the vaccine spike protein binds to the ACE2 receptor, it might disrupt the inner ear function, causing hearing loss. Because most of the tinnitus cases showed no hearing loss, and hearing loss is only weakly correlated with COVID-19 vaccination-related tinnitus, inner ear dysfunction is unlikely to be the only mechanism of COVID-19 vaccination-related tinnitus. *In vitro* translation of the mRNA vaccines is subject to ribosomal frameshifting resulting in off-target proteins (Mulroney et al., 2023). The impact of the off-target protein remains to be determined. However, since non-mRNA COVID-19 vaccines are also associated with increased risk of tinnitus, off-target production in unlikely to be the main cause of COVID-19 vaccination-related tinnitus.

### The risk-benefit balance

Findings of the present study suggests that COVID-19 vaccination increases the risk of tinnitus. The risk is small, resulting in approximately 47–70 VAERS reports per million full vaccinations, ranking the 44th most common complaints following COVID-19 vaccination in the VAERS database. Please note that tinnitus and other adverse events are likely underreported in the VAERS database (Block, 2023). The real frequency of COVID-19 vaccination-related tinnitus remains to be determined. It is unclear how many of the cases ended with symptom resolution either spontaneously or after medical treatment. However, tinnitus in some survey respondents did not resolve over a period up to 250 days. COVID-19 vaccination-related tinnitus may also be comorbid with other neurological and psychiatric symptoms, contributing to the hesitation in accepting vaccination in some people. Thus, it is important to consider the risk and benefit of vaccination. U.S. Center for Disease Control reported approximately 40% of adults have had COVID-19 and 19% of them had long COVID—i.e., symptoms similar to the COVID-19 vaccine adverse effects. Compared to COVID-19 vaccination, COVID-19 infection presents a much higher risk of developing tinnitus—4.5% of the infected population developed tinnitus (Jafari et al., 2022). Vaccination reduces the rate of infection and severity of the symptoms of COVID-19, potentially reducing the overall risk of developing tinnitus and other neurological and psychiatric diseases. It is also important to note that metabolic disorders, a potential risk factor for COVID-19 vaccination-related tinnitus, is also a major risk factor for severe COVID-19 symptoms.

## Data Availability

The raw data supporting the conclusion of this article will be made available by the authors, without undue reservation.
